# A Temperature Plasmonic Sensor Based on a Side Opening Hollow Fiber Filled with High Refractive Index Sensing Medium

**DOI:** 10.3390/s19173730

**Published:** 2019-08-29

**Authors:** Lei Zhao, Haixia Han, Nannan Luan, Jianfei Liu, Li Song, Yongsheng Hu

**Affiliations:** 1 Tianjin Key Laboratory of Electronic Materials and Devices, School of Electronic and Information Engineering, Hebei University of Technology, Tianjin 300401, China; 2 State Key Laboratory of Luminescence and Applications, Changchun Institute of Optics, Fine Mechanics and Physics, Chinese Academy of Sciences, Changchun 130033, China

**Keywords:** temperature sensors, surface plasmon resonance, microstructured optical fibers, fiber optics sensors

## Abstract

A surface plasmon resonance temperature sensor based on a side opening hollow-core microstructured optical fiber is proposed in this paper. This design employs a gold nanowire to excite the plasmon mode, and can be easily filled with the sensing medium through the side opening of the fiber, which not only simplifies the fabrication of the sensor but can also use the high refractive index sensing medium. The coupling characteristics, sensing performance and fabrication tolerance of the sensor are analyzed by using the finite element method. The simulation results indicate that the maximum sensitivity is 3.21 nm/°C for the *x*-polarized core mode in the temperature range of 13.27–50.99 °C, and 4.98 nm/°C for the *y*-polarized core mode in the temperature range of 14.55–51.19 °C, when benzene is used as the sensing medium. The sensor also shows a good stability in the range of ±10% fabrication tolerance.

## 1. Introduction

Surface plasmon resonance (SPR) has high sensitivity to refractive index (RI) changes, which makes it useful for the detection of physical, chemical and biological quantities [1,2,3,4,5,6,7,8]. Recently, microstructured optical fibers (MOF) were actively studied in SPR sensing [5,6,8,9,10,11]. In comparison with other configurations such as prisms and conventional optical fibers [1,2,6,8], benefits of the MOFs are that they can realize desirable guiding properties and the convenience of reasonable mechanical strength [2,5,8,9,10,11]. In most MOF-SPR sensors, to implement SPR sensing for liquid samples, the cladding holes of the MOFs are basically required to be coated with the metal films and infused with the samples [2,5,8,9,10,11]. Moreover, by replacing the liquid sample with a large thermo-optic coefficient sensing medium, these MOF-SPR sensors can be utilized for temperature sensing [12,13,14]. Compared with other types of fiber temperature sensors [15,16,17,18,19,20,21,22], the advantages of MOF-SPR temperature sensors are that they can achieve higher sensitivity and can tune the sensitivity and sensing range by changing the sensing medium [12,13,14].

In these reported MOF-SPR temperature sensors [12,13,14], the sensing medium are filled into the cladding holes of the MOF. In order to maintain the light guiding mechanism of the total reflection, the highest RI of the sensing medium must be lower than the RI of the fiber material. For example, it is usually less than 1.42 in fibers made of silica whose RI is assumed to be 1.45 [12,13,14]. Therefore, these SPR temperature sensors cannot employ some high RI sensing media which have a high thermal coefficient, such as toluene, benzene, liquid crystal materials, etc. [23,24]. Besides, the air hole diameter of the MOF is usually a few microns [12,13,14]. Coating the walls of such small holes with the metal layer and filling them with the sensing medium requires high precision and complicated processes. 

To solve the problems mentioned above, we designed a SPR temperature sensor based on a side opening hollow MOF in this paper. So far, the hollow fibers filled with the liquid have demonstrated that they can work effectively when the RI of the liquid is higher than that of the fiber material [25,26]. Additionally, the open-structured MOFs, such as exposed-core MOFs, have also been studied and the liquids can easily be filled into the sensing region [27,28,29,30,31,32]. This design combines the advantages of the hollow fibers and open-structured MOFs, and thus can employ high RI sensing media without the complex filling process. Moreover, the sensor adopts a gold nanowire to excite the surface plasmon polaritons (SPP) mode, which avoids the complex fabrication of the metal coating. We used the COMSOL Multiphysics software to analyze the SPR character and sensing performance of the sensor, and we also discuss sensor stability in the range of ±10% fabrication tolerance.

## 2. Structure and Principle

**Figure 1:**
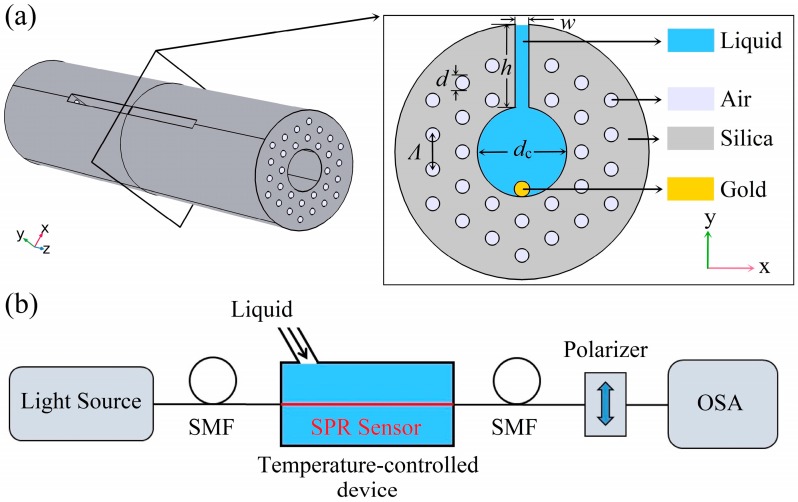
(**a**) Schematic diagram of the surface plasmon resonance (SPR) sensor based on a side opening hollow fiber and (**b**) experimental setup diagram of the proposed SPR sensor for temperature sensing.

As shown in Figure 1a, the hollow core of the MOF-SPR sensor is surrounded by a double-layered hexagonal array of air holes. The sensing medium and the gold nanowire are filled in the hollow core of the fiber. A slot is cut along the length of the fiber to connect the outside and the hollow core, which can accelerate the filling process of the sensing medium. The slot can be fabricated by some mature technologies such as focused ion beam milling [33,34] or femtosecond laser micromachining [35,36]. Both ends of the sensor can be connected to the single mode fiber (SMF) to access the sensing system for practical experimental testing, as shown in Figure 1b.

The electromagnetic mode of the MOF is studied by finite element method (FEM). The sensor structure parameters are shown in Figure 1, where the lattice constant (*Λ*) of the MOF is 2 μm. Both the diameter of the air holes (*d*) and the width of the slot (*w*) are 0.4 *Λ*, and the diameter of the gold nanowire (*d*_g_) is 200 nm. The opening depth (*h*) and the core diameter (*d*_c_) of the MOF are 2 *Λ* and 2.6 *Λ*, respectively. Here, we ignore the material dispersion of the fused silica RI, assuming it is 1.45. The RI of air is assumed to be 1, and the permittivity (*ϵ*_DL_) of the gold can be calculated by the Drude-Lorentz model as [37]:

(1) $$\epsilon_{\mathrm{DL}}\left(\omega \right)=\epsilon_{\infty }-\frac{\omega_{D}^{2}}{\omega \left(\omega +i\gamma_{D}\right)}-\frac{\Delta \epsilon \cdot \Omega_{L}^{2}}{\left(\omega^{2}-\Omega_{L}^{2}\right)+i\Gamma_{L}\omega }$$

where the parameters and values can be found in Reference [37]. The perfectly matched layer (PML) and the triangular sub-domain are used to match the outmost layer and discretize the computation region, respectively.

**Figure 2:**
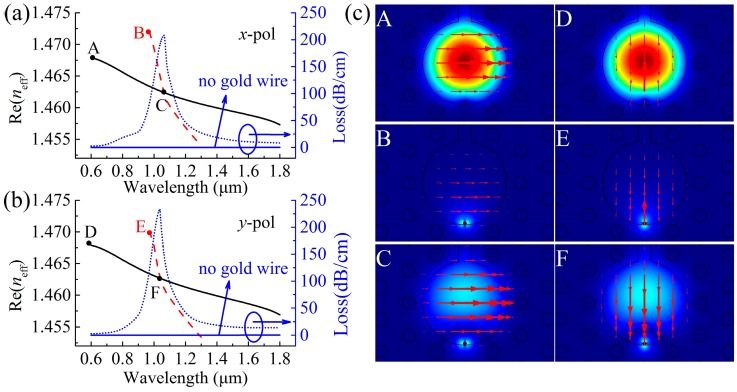
(**a**) Dispersion relations of the *x*-polarized core mode and surface plasmon polaritons (SPP) mode, losses as a function of wavelength for the *x*-polarized core mode, (**b**) dispersion relations of the *y*-polarized core mode and SPP mode, losses as a function of wavelength for the *y*-polarized core mode when the *n* is 1.47, and (**c**) electric field distributions of the relevant modes where the red arrows show the polarization direction of the electric field.

In order to satisfy the conditions for total reflection and ensure the sensor can work normally, the sensing medium RI used here must be higher than the silica RI. In Figure 2, we present the real part of the *n*_eff_ [Re(*n*_eff_)] curve, loss spectra and the electric field distribution of the correlation mode of the sensor when the sensing medium RI (*n*) is 1.47. The red dashed lines in Figure 2a,b denote the Re(*n*_eff_) of the *x*- and *y*-polarized SPP modes excited on the surface of the nanowire. The black solid lines and blue dashed lines in Figure 2a,b represent the Re(*n*_eff_) and loss spectra of the *x*- and *y*-polarized core modes. As a result, the *x*-polarized resonance peak is at 1.066 μm (phase matching point C) and the *y*-polarized resonance peak is at 1.037 μm (phase matching point F). At non-resonance wavelengths, the *x*- and *y*-polarized core modes are well limited in the core region as shown by patterns A and D in Figure 2c, while the *x*- and *y*-polarized SPP modes are well limited on the surface of the nanowire, as shown by patterns B and E in Figure 2c. At resonance wavelength, as seen from patterns C and F in Figure 2c, the *x*- and *y*-polarized core modes show syncretic patterns which means that they are coupled to the *x*- and *y*-polarized SPP modes respectively, and thus generating the resonance peaks in the loss spectra. If the *n* is changed by the temperature, the Re(*n*_eff_) of the relevant mode is also changed accordingly, and causes the shift of the resonance peak. Therefore, this mechanism can be used to detect the temperature changes.

## 3. Results and Discussion

### 3.1. Sensing Performance

Here, we adopt benzene as the sensing medium, and its dispersion equation is given as [23]:

(2) $$n_{0}=1.475922+\frac{9671.57}{\lambda^{2}}-5.2538\times \frac{10^{8}}{\lambda^{4}}+8.5442\times \frac{10^{13}}{\lambda^{6}}-2.6163\times \frac{10^{18}}{\lambda^{8}}$$

**Figure 3:**
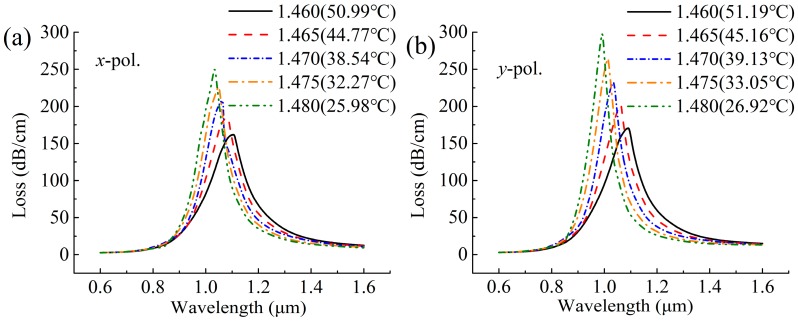
Losses as a function of wavelength for the (**a**) *x*-polarized and (**b**) *y*-polarized core modes at different *n* (temperatures).

The temperature-induced change of the benzene RI (n) is evaluated by [12,13,14]:

(3) $$n=n_{0}+\frac{dn}{dT}\times \left(T-T_{0}\right)$$

where the thermal coefficient (dn/dT) of the benzene is 7.594 × 10^−4^/°C at the reference temperature T_0_ = 20 °C [23]. Here, in order to simplify the calculation method, the thermal coefficients of the gold and the silica are negligible because they are much lower than liquids. Therefore, temperature changes can be assumed to change the n only. To investigate the sensing performance of the proposed sensor for temperature sensing, in Figure 3 we present the loss spectra of the x- and y-polarized core modes at different n affected by temperature changes. Here, as we observe the temperature (T) decreasing, the position of the resonance peak shifts to a shorter wavelength, which is consistent with the phenomena of the hollow MOF-SPR sensors [25,26]. The temperature sensitivity is calculated by [12,13,14].

(4) $$S\left[nm/℃\right]=\Delta \lambda_{peak}/\Delta T$$

**Figure 4:**
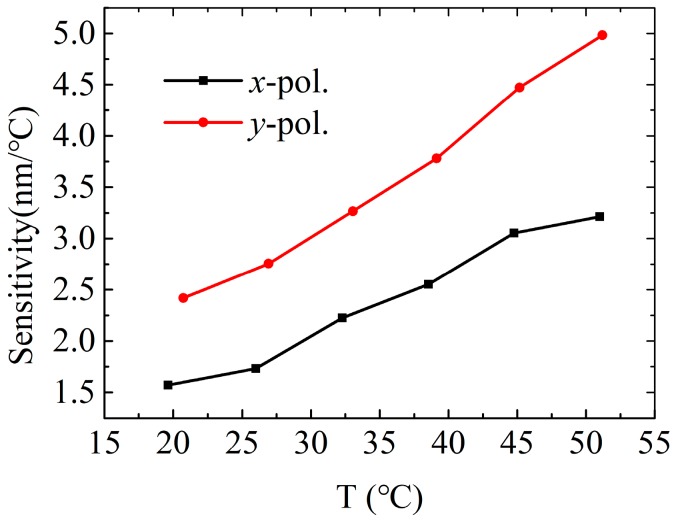
Temperature sensitivities of the *x*-polarized and *y*-polarized core modes at different temperatures.

Figure 4 shows the temperature sensitivities of the *x*- and *y*-polarized core modes at different temperatures. In general, the *y*-polarized sensitivities are higher than the *x*-polarized sensitivities, and they are gradually increasing with increasing temperatures. The maximum *x*- and *y*-polarized sensitivities are 3.21 nm/°C in the detection range of 13.27–50.99 °C and 4.98 nm/°C in the detection range of 14.55–51.19 °C, respectively. This value is much higher than that of other fiber optic temperature sensors, as shown in Table 1. Note that the sensitivity and detection range of the proposed sensor can be changed if using other sensing media. For example, the maximum sensitivities are 2.21 nm/°C for the *x*-polarized core mode in the temperature range of 13.27–59.15 °C, and 3.40 nm/°C for the *y*-polarized core mode in the temperature range of 14.55-59.38 °C, when we employ the toluene as the sensing medium whose thermal coefficient and dispersion equation can be found in Reference [23]. Furthermore, they also can be adjusted to a desirable value by using some tunable RI sensing media such as liquid crystal [24] and liquid mixture [12].

### 3.2. Fabrication Tolerance

For the nanowire filled MOF-SPR temperature sensor in this paper, some fabrication tolerances that occur in actual manufacturing may have an impact on the sensing performance. Therefore, in the next section, we discuss the effect of fabrication tolerances in the range of ±10%. 

**Figure 5:**
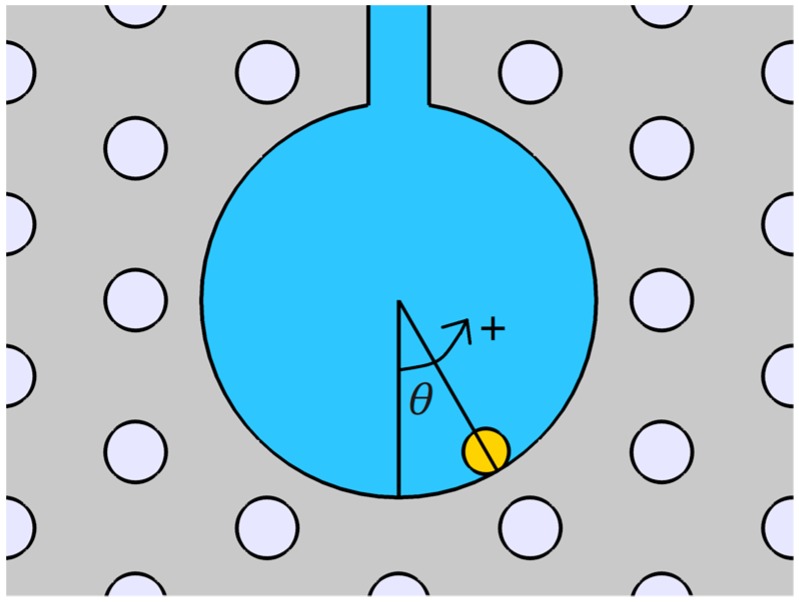
Schematic of the fiber hole filled with the gold nanowire.

**Figure 6:**
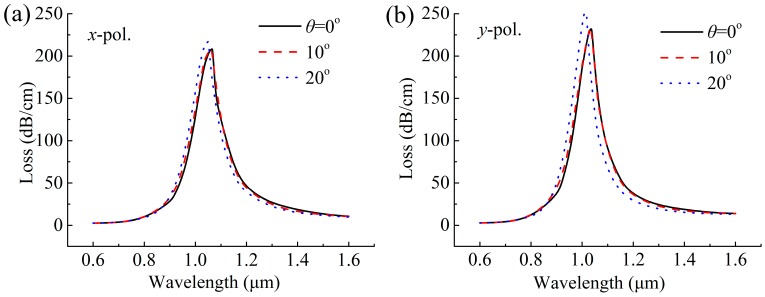
Losses as a function of wavelength for the (**a**) *x*-polarized and (**b**) *y*-polarized core modes at *n* = 1.47, with varying nanowire positions (*θ*).

The first fabrication tolerance to be considered is the effect of gold nanowire positions on the SPR spectra. This manufacturing method of filling the nanowire directly into the MOF hollow core simplifies the manufacturing process compared to the previously mentioned coating process [9,10,11,12,13]. However, it is hard to keep the position of the nanowire in the MOF hole stationary. Here, we only consider the effect of the nanowire at the bottom region of the hole under gravity, as shown in Figure 5. The rotation angle (*θ*) is used to describe the position of the nanowire, and the case where *θ* is positive indicates that the nanowire is deflected to the right. Because of symmetry, only the case where *θ* is positive is considered. When *θ* changes from 0° to 20° in Figure 6, the *x*- and *y*-polarized resonance peaks move to shorter wavelengths and the moving distance is very small, which has a smaller effect on the corresponding sensitivity, especially in the range of 0° to 10°.

**Figure 7:**
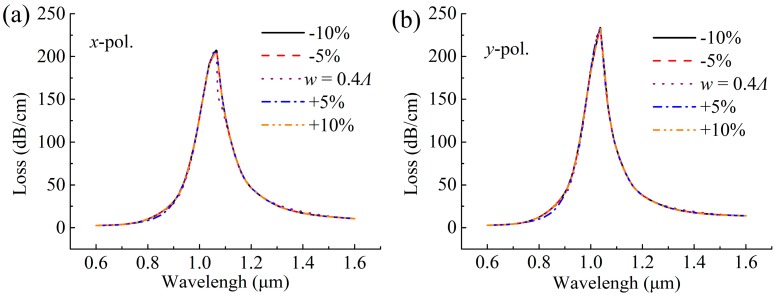
Losses as a function of wavelength for the (**a**) *x*-polarized and (**b**) *y*-polarized core modes at *n* = 1.47, with varying width of the slot (*w*).

**Figure 8:**
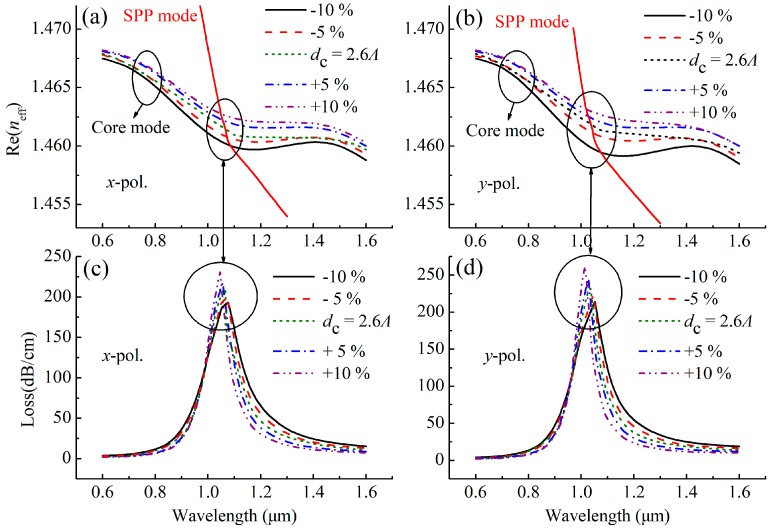
(**a**) Dispersion relations of the *x*-polarized core mode and SPP mode, (**b**) dispersion relations of the *y*-polarized core mode and SPP mode, (**c**) losses as a function of wavelength for the *x*-polarized core mode and (**d**) losses as a function of wavelength for the *y*-polarized core mode at *n* = 1.47, with varying core diameter (*d*_c_).

**Figure 9:**
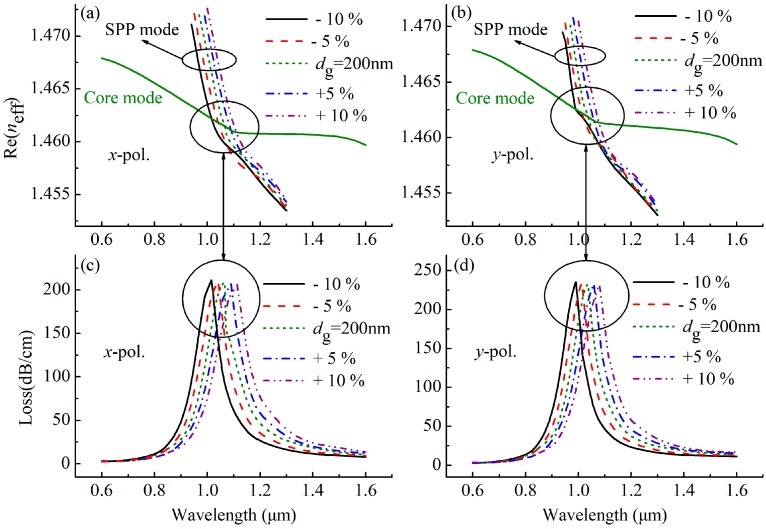
(**a**) Dispersion relations of the *x*-polarized core mode and SPP mode, (**b**) dispersion relations of the *y*-polarized core mode and SPP mode, (**c**) losses as a function of wavelength for the *x*-polarized core mode and (**d**) losses as a function of wavelength for the *y*-polarized core mode at *n* = 1.47, with varying gold nanowire (*d*_g_).

For other fabrication tolerances, the effect of the parameters *w* = 0.4 *Λ*, *d*_c_ = 2.6 *Λ* and *d*_g_ = 200 nm on the SPR spectra at *n* = 1.47 are shown in Figure 7, Figure 8 and Figure 9. As shown in Figure 7, the position of the resonance peak is less affected by *w*, indicating that the sensor has good stability in the range of ±10% fabrication tolerance of *w*. However, as the *d*_c_ increases in Figure 8, the resonance peak shifts toward the short wavelength direction, which is because the increase in *d*_c_ causes the Re(*n*_eff_) of the core mode to become larger, as shown in Figure 8a,b, resulting in the intersection of the Re(*n*_eff_) curves of the core mode and the SPP mode, that is, the peak shifts to the short wavelength direction. The increase in *d*_c_ also reduces the loss of the core mode, which can make the resonance peak width narrower, as seen from Figure 8c,d, and leads to a better resolution. Figure 9 shows the effect of *d*_g_ on the loss spectra of the proposed sensor, where *d*_g_ changes in the range of –10% to 10%, and the resonance peak shifts toward the long wavelength direction as *d*_g_ increases. This is because the increases in *d*_g_ increases the Re(*n*_eff_) of the SPP mode, as shown in Figure 9a,b, and thus leads to the intersection (the peak) which moves toward the long wavelength, as seen from Figure 9c,d. In general, when the gold nanowire is within ±20° deflection range and the sensor parameters (*w*, *d*_c_, and *d*_g_) are within ± 10% tolerances, the SPR phenomenon is relatively stable, indicating that the sensor can work normally.

## 4. Conclusions

This paper describes a SPR temperature sensor based on a side opening hollow-core MOF, which improves the problems of most SPR temperature sensors which cannot use high RI sensing media and need complex coating and filling processes. Taking benzene as the sensing medium for an example, the maximum sensitivity of the sensor is 4.98 nm/°C for the *y*-polarized core mode in the temperature range of 14.55–51.19 °C, while maximum sensitivity and temperature range can be adjusted by changing the sensing medium. In addition, the sensor also shows good stability in the range of ± 10% fabrication tolerances. This sensor design can be used not only for temperature sensing with the high RI sensing media, but also for the real-time sensing of high RI samples.

## Figures and Tables

**Table 1:** Sensitivity comparison of various fiber optic temperature sensors.

Fiber Structure	Sensitivity (nm/°C)	Temperature Range (°C)	Ref.
Fabry-Pérot cavity	0.0846	20–100	[17]
Fiber Bragg grating	0.172	30–65	[18]
Mach-Zehnder interferometer	0.014	10–70	[19]
Liquid sealed PCF	0.166	23.7–66.1	[20]
Modal interferometer	0.0926	28–51	[21]
FFPI	0.014	−79–70	[22]
MOF-SPR	0.72	0–50	[13]
Side opening hollow fiber-SPR	4.98	14.55–51.19	This work

## References

[1] Sharma A.K., Pandey A.K., Kaur B. (2018). A Review of advancements (2007–2017) in plasmonics-based optical fiber sensors. Opt. Fiber Technol..

[2] Klantsataya E., Jia P., Ebendorff-Heidepriem H., Monro T.M., François A. (2016). Plasmonic fiber optic refractometric sensors: from conventional architectures to recent design trends. Sensors.

[3] Zhao Y., Lei M., Liu S., Zhao Q. (2018). Smart hydrogel-based optical fiber SPR sensor for pH measurements. Sens. Actuators B Chem..

[4] Zhao Y., Wu Q., Zhang Y. (2018). Theoretical analysis of high-sensitive seawater temperature and salinity measurement based on C-type micro-structured fiber. Sens. Actuators B Chem..

[5] Rifat A.A., Ahmed R., Yetisen A.K., Butt H., Sabouri A., Mahdiraji G.A., Yun S.H., Adikan F.M. (2017). Photonic crystal fiber based plasmonic sensors. Sens. Actuators B Chem..

[6] Zhao Y., Deng Z., Li J. (2014). Photonic crystal fiber based surface plasmon resonance chemical sensors. Sens. Actuators B Chem..

[7] Singh P. (2016). SPR biosensors: Historical perspectives and current challenges. Sens. Actuators B Chem..

[8] Aruna Gandhi M.S., Chu S., Senthilnathan K., Ramesh Babu P., Nakkeeran K., Li Q. (2019). Recent advances in plasmonic sensor-based fiber optic probes for biological applications. Appl. Sci..

[9] Hassani A., Skorobogatiy M. (2006). Design of the microstructured optical fiber-based surface plasmon resonance sensors with enhanced microfluidics. Opt. Express..

[10] Hautakorpi M., Mattinen M., Ludvigsen H. (2008). Surface-plasmon-resonance sensor based on three-hole microstructured optical fiber. Opt. Express.

[11] Zhang Y., Xia L., Zhou C., Yu X., Liu H., Liu D., Zhang Y. (2011). Microstructured fiber based plasmonic index sensor with optimized accuracy and calibration relation in large dynamic range. Opt. Commun..

[12] Luan N., Wang R., Lu Y., Yao J. (2014). Simulation of surface plasmon resonance temperature sensor based on liquid mixture-filling microstructured optical fiber. Opt. Eng..

[13] Peng Y., Hou J., Huang Z., Lu Q. (2012). Temperature sensor based on surface plasmon resonance within selectively coated photonic crystal fiber. Appl. Opt..

[14] Luan N., Wang R., Lu Y., Yao J. (2014). Surface plasmon resonance temperature sensor based on photonic crystal fibers randomly filled with silver nanowires. Sensors.

[15] Schena E., Tosi D., Saccomandi P., Lewis E., Kim T. (2016). Fiber optic sensors for temperature monitoring during thermal treatments: An overview. Sensors.

[16] Ramakrishnan M., Rajan G., Semenova Y., Farrell G. (2016). Overview of fiber optic sensor technologies for strain/temperature sensing applications in composite materials. Sensors.

[17] Liu G., Han M., Hou W. (2015). High-resolution and fast-response fiber-optic temperature sensor using silicon Fabry-Pérot cavity. Opt. Express.

[18] Hu T., Zhao Y., Song A. (2016). Fiber optic SPR sensor for refractive index and temperature measurement based on MMF-FBG-MMF structure. Sens. Actuators B Chem..

[19] Bai Y., Yin B., Liu C., Liu S., Lian Y., Jian S. (2014). Simultaneous measurement of refractive index and temperature based on NFN structure. IEEE Photon. Technol. Lett..

[20] Qiu S., Chen Y., Xu F., Lu Y. (2012). Temperature sensor based on an isopropanol-sealed photonic crystal fiber in-line interferometer with enhanced refractive index sensitivity. Opt. Lett..

[21] Zhao Y., Cai L., Li X. (2015). High sensitive modal interferometer for temperature and refractive index measurement. IEEE Photon. Technol. Lett..

[22] Li X., Lin S., Liang J., Zhang Y., Oigawa H., Ueda T. (2011). Fiber-optic temperature sensor based on difference of thermal expansion coefficient between fused silica and metallic materials. IEEE Photonics J..

[23] Samoc A. (2003). Dispersion of refractive properties of solvents: Chloroform, toluene, benzene, and carbon disulfide in ultraviolet, visible, and near-infrared. J. Appl. Phys..

[24] Du C., Wang Q., Zhao Y. (2018). Electrically tunable long period gratings temperature sensor based on liquid crystal infiltrated photonic crystal fibers. Sens. Actuators B Phys..

[25] Liu B.H., Jiang Y.X., Zhu X.S., Tang X.L., Shi Y.W. (2013). Hollow fiber surface plasmon resonance sensor for the detection of liquid with high refractive index. Opt. Express.

[26] Luan N., Yao J. (2016). High refractive index surface plasmon resonance sensor based on a silver wire filled hollow fiber. IEEE Photonics J..

[27] Klantsataya E., François A., Ebendorff-Heidepriem H., Hoffmann P., Monro T.M. (2015). Surface plasmon scattering in exposed core optical fiber for enhanced resolution refractive index sensing. Sensors.

[28] Warren-Smith S.C., Ebendorff-Heidepriem H., Foo T.C., Moore R., Davis C., Monro T.M. (2009). Exposed-core microstructured optical fibers for real-time fluorescence sensing. Opt. Express.

[29] Luan N., Yao J. (2015). Surface plasmon resonance sensor based on exposed-core microstructured optical fiber placed with a silver wire. IEEE Photonics J..

[30] Gómez-Cardona N.D., Reyes-Vera E., Torres P. (2018). Multi-plasmon resonances in microstructured optical fibers: Extending the detection range of SPR sensors and a multi-analyte sensing technique. IEEE Sens. J..

[31] Wang X.-Z., Wang Q. (2019). Theoretical Analysis of a Novel Microstructure Fiber Sensor Based on Lossy Mode Resonance. Electronics.

[32] Yang X., Lu Y., Wang M., Yao J. (2015). An exposed-core grapefruit fibers based surface plasmon resonance sensor. Sensors.

[33] Wang F., Yuan W., Hansen O., Bang O. (2011). Selective filling of photonic crystal fibers using focused ion beam milled microchannels. Opt. Express.

[34] Martelli C., Olivero P., Canning J., Groothoff N., Gibson B., Huntington S. (2007). Micromachining structured optical fibers using focused ion beam milling. Opt. Lett..

[35] van Brakel A., Grivas C., Petrovich M.N., Richardson D.J. (2007). Micro-channels machined in microstructured optical fibers by femtosecond laser. Opt. Express.

[36] Hensley C.J., Broaddus D.H., Schaffer C.B., Gaeta A.L. (2007). Photonic band-gap fiber gas cell fabricated using femtosecond micromachining. Opt. Express.

[37] Vial A., Grimault A.S., Macías D., Barchiesi D., de la Chapelle M. (2005). Improved analytical fit of gold dispersion: application to the modeling of extinction spectra with a finite-difference time-domain method. Phys. Rev. B..

